# Effect of a culturally tailored educational intervention on women’s knowledge and attitudes toward cervical cancer screening: a single-group pre-post study

**DOI:** 10.4069/whn.2026.02.28.1

**Published:** 2026-03-31

**Authors:** Rania Najeh Abuzeyad, Nahla Al Ali, Diala Al Twalbeh

**Affiliations:** 1 Department of Community and Mental Health, Faculty of Nursing, Jordan University of Science and Technology, Irbid, Jordan; 2 Department of Adult Health Nursing, College of Nursing, Sultan Qaboos University, Muscat, Oman

**Keywords:** Cervical cancer, Health education, Health knowledge, attitudes, practice, Papanicolaou test, Women’s health

## Abstract

**Purpose:**

This study aimed to evaluate the effectiveness of a culturally tailored educational intervention on Jordanian women’s knowledge and attitudes toward cervical cancer screening.

**Methods:**

A single-group pre-post intervention study was conducted at two primary healthcare centers in Jordan. Sixty women aged 25 to 45 years were recruited using convenience sampling. The intervention consisted of 10 face-to-face group sessions (approximately 60 minutes each) delivered over 4 weeks by trained female nurse educators using interactive, culturally sensitive materials. Participants completed an Arabic-language questionnaire assessing cervical cancer knowledge, attitudes toward screening, perceived barriers, and prior screening history. Wilcoxon signed-rank tests assessed pre-post changes, while nonparametric comparisons and Spearman correlations examined associations with demographic characteristics.

**Results:**

At baseline, 51.7% of participants had heard of the Papanicolaou (Pap) test, 13.3% had ever undergone a Pap test, and 10.0% intended to be screened within the next 3 years. The most frequently reported barriers were embarrassment (86.7%), fear of a negative result (85.0%), and discomfort (83.3%). Knowledge increased significantly (median, 3.00 to 16.00; Z=−6.73, *p*<.001; Cohen’s d=0.87), and attitudes improved (median, 27.00 to 36.00; Z=−6.62, *p*<.001; d=0.85) following the intervention. Higher income and prior awareness of the Pap test were significantly associated with greater post-intervention knowledge. Higher income and older age were also significantly associated with more favorable attitudes (*p*<.05).

**Conclusion:**

A culturally tailored educational program delivered in primary care produced substantial improvements in women’s knowledge and attitudes. Integrating structured cervical cancer education into routine women’s health services may support informed screening decisions and strengthen cervical cancer prevention efforts in Jordan.

## Introduction

Cervical cancer is largely preventable through human papillomavirus (HPV) vaccination and effective screening; however, it remains an important cause of morbidity and mortality among women worldwide. In 2020, an estimated 604,000 new cases and 342,000 deaths were reported globally, with approximately 90% of cases and deaths occurring in low- and middle-income countries [1,2].

To accelerate progress, the World Health Organization launched a global strategy to eliminate cervical cancer as a public health problem, establishing targets for HPV vaccination, screening, and treatment (the 90-70-90 targets). Achieving these targets requires not only the availability of services but also informed participation in screening [3].

International guidelines recommend regular cervical cancer screening for women in mid-adulthood. Women aged 25 to 65 years can be screened using primary HPV testing every 5 years; when HPV testing is unavailable, co-testing (HPV testing plus cytology) every 5 years or cytology (Papanicolaou [Pap] test) every 3 years are acceptable alternatives [4,5].

In Jordan, cervical cancer screening is not organized at the national level and is largely opportunistic and clinic-based. Published evidence indicates low uptake, with many eligible women reporting that they have never been screened. National data also report relatively low incidence and mortality (3.4 and 1.7 per 100,000 women, respectively, in 2020); nevertheless, preventable cases and late presentation remain concerns [5-8].

Low participation in cervical cancer screening is consistently associated with limited knowledge, low perceived susceptibility, and sociocultural barriers. Studies conducted in Jordan have reported embarrassment during pelvic examinations [6,9], fear of a negative result [6], fear of pain or discomfort and misconceptions about harm [9], limited spousal support [9], and limited discussion or recommendation from healthcare providers [6] as key barriers to Pap test uptake.

Education represents a modifiable leverage point: well-designed interventions can improve women’s knowledge and attitudes regarding cervical cancer prevention and screening and may reduce perceived barriers [10-12]. However, culturally tailored, primary-care-based programs in Jordan remain limited, and evidence from rigorous pre-post evaluations is needed. The present study was designed to address this gap.

The present study aimed to evaluate the impact of an educational intervention on Jordanian women’s knowledge and attitudes regarding cervical cancer and cervical cancer screening.

The study sought to answer the following questions:

1. What are the knowledge, attitudes, and practices related to cervical cancer and cervical cancer screening among women attending primary healthcare centers in Jordan?

2. What are the sources of women’s prior knowledge about cervical cancer and cervical cancer screening?

3. What barriers do Jordanian women encounter when undergoing a Pap test?

4. What is the effect of cervical cancer education programs on women’s knowledge and attitudes toward cervical cancer and cervical cancer screening?

5. How do women’s characteristics influence their knowledge and attitudes toward cervical cancer and cervical cancer screening?

## Methods

**Ethics statement:** Approval was granted by the Ethics Committee of Jordan University of Science and Technology (No. 3/149/2022, dated 13/06/2022) and the Ethics Committee of the Jordan Ministry of Health (No. 249/2022/REC/MOH, dated 17/08/2022). Written informed consent was obtained from all participants. Participation was voluntary, and participants could withdraw at any time without affecting their care. All responses were collected anonymously and kept confidential. No monetary incentives were provided; educational materials and information about local screening services were provided free of charge.

### Research design

This study used a single-group pre-post intervention design and was conducted at two primary healthcare centers in Jordan.

### Sample and sampling

Convenience sampling was used to recruit women aged 25 to 45 years who attended the selected primary healthcare centers affiliated with the Irbid Health Directorate in northern Jordan. The age range of 25 to 45 years was selected because it represents the target group for cervical cancer screening in Jordan. Inclusion criteria were as follows: being married, being able to understand and engage with the educational materials, willingness to participate in all intervention sessions, and availability for post-intervention evaluation. Women were excluded if they were outside the specified age range, had a previous diagnosis of cervical cancer, or were undergoing treatment for cervical cancer (chemotherapy or radiotherapy).

Sample size was estimated using G*Power 3.0.10. Based on a medium effect size (Cohen’s d=0.43), 80% power, and a significance level of α=.05 (two-tailed), the minimum required sample size was 45 participants. A total of 60 participants were recruited.

Two comprehensive healthcare centers were purposively selected as study sites. After ethical and institutional approvals were obtained, the researchers collaborated with healthcare personnel to identify women who met the inclusion criteria. Eligible women attending the maternal and child health departments during the recruitment period were approached by a research assistant, informed about the study objectives and procedures, and invited to participate.

### Instruments

Data were collected using a structured Arabic questionnaire. The instrument was translated into Arabic by an experienced medical/health translator and was subsequently reviewed by a panel of bilingual experts, including a professor of nursing with expertise in women’s health, to confirm semantic equivalence, clarity, and cultural relevance. The items were culturally adapted for Jordanian women (e.g., simplified wording and use of locally familiar terms such as Pap test) while maintaining the original meaning. A pilot test with a small group of women confirmed clarity and acceptability, and minor wording adjustments were made before data collection.

#### Knowledge, Attitude, and Practice scale

The Knowledge, Attitude, and Practice (KAP) scale included 35 items: 18 knowledge items (score range, 0–18), nine attitude items rated on a 5-point Likert scale (total score, 9–45), and eight practice items [13]. Higher scores indicate greater knowledge, more favorable attitudes, and more screening-related practices. The overall Cronbach’s α coefficient for the original scale was reported as .90, whereas the coefficients for the KAP subscales were 0.80, 0.82, and 0.93, respectively [13]. In the present study, internal consistency was high for each subscale (Cronbach’s α: knowledge=.95; attitude=.80; practice=.87).

#### Perceived barriers scale

The perceived barriers scale included five yes/no items (yes=1, no=0), with yes responses indicating greater perceived barriers to screening [14]. These items assessed discomfort, embarrassment, fear of a negative result, concern about the cost of the Pap test, and desire for a Pap test.

#### General characteristics

The researcher developed 17 items to assess women’s demographic and reproductive characteristics on the basis of the literature. The items covered age, educational level, marital status, health insurance status, employment status, monthly income, age at first pregnancy, age at first marriage, age at menarche, parity, family planning practices, time since the last visit to a gynecologist, awareness of the Pap test, sources of information, preferred provider for the Pap test, future intention to undergo Pap testing, and family history of cervical cancer.

### Data collection procedure

Managers of the selected healthcare centers were informed about the study’s purpose and the structure of the planned intervention. Participants completed the baseline questionnaire immediately before the first session and completed the post-test questionnaire after the final session. The researcher collected the completed questionnaires and was available to answer questions and provide clarification during data collection.

### Intervention

The educational intervention on cervical cancer was designed as a structured, culturally sensitive program grounded in the current literature [11,15] and aligned with the American Society of Clinical Oncology [16]. To ensure cultural relevance in the Jordanian context, the program content and delivery explicitly addressed barriers reported in prior local and regional research, particularly inadequate knowledge, fear of adverse or positive results, and embarrassment or modesty related to pelvic examination, including preference for female providers and the influence of family or spousal support on screening decisions. The culturally tailored educational intervention was delivered face-to-face in small groups (5–7 participants per group) over 4 weeks. It comprised 10 sessions (approximately 60 minutes each) delivered by two trained female nurse educators using interactive strategies such as group discussions, case scenarios, skills practice, mapping activities, and role-play. Practical cultural components were integrated across sessions by (1) normalizing embarrassment and stigma through guided discussion and anonymous question-and-answer sessions, (2) addressing fear of results by explaining common outcomes and follow-up pathways, and (3) strengthening communication skills and social support (e.g., how to request screening, how to discuss screening with family members, and how to communicate preferences to providers). Content addressed cervical cancer risk, prevention, screening procedures, and common misconceptions (Table 1).

To ensure intervention fidelity, the facilitators followed a standardized session guide and used a brief checklist to promote consistent delivery across both sites. Participants received session reminders and were encouraged to attend all sessions and engage actively in the interactive teaching strategies (e.g., group discussions and question-and-answer segments). The principal investigator monitored fidelity through periodic observation of sessions to ensure content completeness and consistent messaging across both primary healthcare centers.

### Data analysis

Data were analyzed using IBM SPSS ver. 26 (IBM Corp., Armonk, NY, USA). Because several variables were not normally distributed, nonparametric tests were used. Wilcoxon signed-rank tests assessed pre-post changes in knowledge and attitude scores, whereas Mann-Whitney U tests, Kruskal-Wallis tests, and Spearman correlations were used to examine associations with demographic characteristics. Effect sizes were reported where appropriate. A significance level of *p*<.05 was used for all statistical tests.

## Results

### Characteristics of the study participants

Table 2 summarizes participant characteristics (n=60). The mean age was 34.88 years (SD=6.33 years). Most participants were married (98.3%), insured (86.7%), and had completed college or university education (63.3%). Mean monthly income was 388.66 Jordanian Dinar (JD) (SD=188.37 JD, approximately 548 USD), which is lower than the national average, based on the mean household income reported in Jordan’s Household Expenditures and Income Survey 2017 [17]. This value is also below the average monthly wage reported by the Department of Statistics for paid employees in economic establishments (544 JD) [18]. At baseline, 51.7% had heard of the Pap test, 13.3% reported ever having undergone a Pap test, and 10.0% intended to be screened within the next 3 years. Most women preferred a female provider for the Pap test (83.3%). Overall, 33.3% of participants (n=20) had never visited a gynecologist.

### Participants’ prior knowledge and barriers

At baseline, 25.6% of participants reported that they had heard only about cervical cancer and screening. Healthcare providers (n=17, 44.7%) and the internet (n=11, 28.9%) were the most commonly reported sources of information. Only a small number of women cited the media, printed materials, workshops, or lectures.

The most commonly reported barriers to cervical cancer screening were embarrassment (86.7%), fear of a negative result (85.0%), and discomfort during the Pap test (83.3%). These findings informed the content emphasis of the educational sessions.

### The impact of education on women’s knowledge and attitudes toward cervical cancer

Wilcoxon signed-rank tests showed significant pre-post improvements in knowledge (median, 3.00 to 16.00; Z=−6.73, *p*<.001, Cohen’s d=0.87) and attitudes (median, 27.00 to 36.00; Z=−6.62, *p*<.001, Cohen’s d=0.85). According to Cohen’s classification, both effects were large (Table 3).

### Post-intervention knowledge and attitudes according to participants’ characteristics

Post-intervention knowledge scores were significantly higher among women who had heard of the Pap test (U=307.00, *p*=.033) and were positively correlated with monthly income (rho=0.28, *p*=.031) (Table 4).

Post-intervention attitudes were more favorable among working women (U=227.00, *p*=.004) and were positively correlated with age (rho=0.30, *p*=.020) and monthly income (rho=0.43, *p*=.001) (Table 5).

## Discussion

The findings indicate that the 10-session, culturally sensitive educational intervention delivered over 4 weeks improved women’s knowledge and attitudes toward cervical cancer and cervical cancer screening. These findings are consistent with the broader literature showing that educational interventions can positively influence cervical cancer–related knowledge and attitudes [19-21], beliefs [20], and, in some cases, screening behavior [22,23]. Importantly, the evidence suggests that effectiveness is driven less by country context than by shared intervention characteristics. Across previous studies, interventions that were structured and clearly sequenced [19,24,25], interactive or community-based in ways that supported discussion and addressed beliefs and misconceptions [20], and delivered by or linked to trained health personnel who could credibly explain screening and respond to concerns tended to report more consistent improvements [21,24]. This pattern is also supported by a systematic review, which concluded that educational approaches can improve screening-related outcomes, although the magnitude of effects varies by intervention design and setting [22]. The present program incorporated these key characteristics, including repeated contact across multiple sessions, interactive strategies (e.g., discussion, case scenarios, and role-play), and delivery by trained female nurse educators, providing a plausible explanation for the observed improvements in both knowledge and attitudes.

Although educational interventions have shown benefits internationally [19-25], this study contributes evidence from a culturally tailored implementation within Jordanian primary healthcare centers. Delivering the program in routine primary healthcare settings using female nurse educators and culturally appropriate messaging may enhance acceptability and feasibility. In addition, the intervention design directly addressed locally relevant barriers, including embarrassment, fear of painful examination or unfavorable results, and limited spousal or family support, which may be particularly important for increasing screening readiness and intention in this context [9].

Although the intervention produced statistically significant improvements in knowledge and attitudes, its practical significance for actual screening behavior remains uncertain. The baseline screening rate in our sample was extremely low: only 13.3% (8 of 60 women) had ever undergone a Pap test. Although the intervention aimed to promote preventive behaviors, this short-term study did not assess post-intervention screening uptake. Furthermore, the persistently high prevalence of sociocultural barriers, such as embarrassment and fear of a negative result, suggests that although women have acquired the necessary information, these deep-seated psychological obstacles are unlikely to be overcome by education alone. Future intervention studies in this context should incorporate behavioral and motivational strategies alongside education and should include longitudinal follow-up to assess actual Pap test uptake and sustained screening intention.

Common obstacles to cervical cancer screening reported across settings include limited knowledge or awareness [26-28], negative beliefs and misconceptions [27,28], and low screening uptake [26-28]. For example, in Ethiopia, low screening uptake co-occurred with substantial gaps in knowledge about cervical cancer prevention [26]. Similar patterns have been documented among women attending primary healthcare centers in Bahrain, where awareness and understanding of Pap testing were limited and misconceptions were common [27], and among women in rural Nepal, where screening uptake was low and awareness of screening was poor [28]. Taken together, these findings are consistent with the barriers observed in the current study and reinforce the need for educational interventions to explicitly address basic factual knowledge, including what screening is, who needs it, and when to initiate it, as well as misconceptions that shape beliefs and reduce perceived need for screening. Accordingly, a practical implication is to integrate clear, repeated messages about screening purpose, eligibility, and follow-up into routine primary healthcare education, using simple language and opportunities for questions.

Embarrassment and modesty concerns are repeatedly identified as barriers that reduce women’s willingness to undergo cervical cancer screening. Systematic reviews indicate that embarrassment is among the most frequently reported barriers across low- and middle-income settings [29], and it has also been highlighted among adolescents and young women [30] and across South Asian contexts [31]. Evidence from Uganda similarly describes embarrassment and discomfort with pelvic examination as reasons for nonattendance [32]. These findings support our results by indicating that embarrassment is a culturally salient barrier and suggesting that interventions should address it as a central implementation issue rather than as a minor attitudinal factor. Previous research in Jordan has also identified inadequate knowledge, fear of unfavorable results, and embarrassment as key barriers to Pap test uptake [8,33]. Thus, screening programs should incorporate strategies that protect privacy and dignity, such as ensuring confidentiality, offering female providers when possible, explaining the procedure step-by-step, and allowing anonymous question-and-answer sessions to normalize concerns, in order to reduce anticipated embarrassment and improve acceptability.

In the present study, fear of receiving unfavorable results emerged as a significant barrier to cervical cancer screening. This barrier has also been reported in other settings, where women have described fear of a cancer diagnosis [34,35], anxiety about follow-up procedures [35], and uncertainty about treatment as reasons for delaying or avoiding screening [34,35]. These findings are consistent with our results and emphasize that educational content should focus not only on risk information but also on what occurs after screening. Therefore, interventions should include clear explanations that abnormal results do not necessarily indicate cancer, should outline follow-up pathways in simple terms, and should provide reassurance about the benefits of early detection paired with skills-based communication, such as what questions to ask and how to request support, in order to reduce anxiety-driven avoidance.

These findings suggest that such obstacles are not merely cognitive gaps that can be addressed by information alone; rather, they reflect deep-seated sociocultural factors related to privacy, social stigma, discomfort with gynecological procedures, and a prevalent preference for a female doctor (83.3%). However, these barriers should also be interpreted within broader system-level constraints, including the availability, affordability, and perceived quality of cervical cancer diagnostic follow-up and treatment services, as well as the clarity of referral pathways and timeliness of access to care, all of which may influence women’s willingness to participate in screening. The persistently high levels of fear therefore highlight the need for multilevel, longer-term strategies that combine culturally sensitive behavioral approaches with service-delivery improvements, such as female-provider availability, privacy-supportive clinic environments, and stronger follow-up and treatment linkages, to facilitate the transition from positive intention and attitudes to actual screening practice.

Our findings indicate that women with higher income had greater knowledge and more favorable attitudes toward cervical cancer and cervical cancer screening. One possible explanation is that women with higher income may have greater access to educational opportunities, healthcare services, and health information, which may increase awareness of cervical cancer and the importance of screening. These findings are consistent with previous studies showing that income is significantly associated with knowledge of cervical cancer and its screening [36-38]. One study also found that women with higher incomes reported significantly more positive attitudes and intentions toward cervical cancer screening [39]. Similarly, our finding that employed women had more positive attitudes toward cervical cancer screening may reflect greater access to healthcare services, greater exposure to workplace health information, and greater financial independence, all of which may facilitate regular screening and higher health literacy.

The finding that older women showed more positive attitudes toward screening may reflect differences in cognitive development, emotional perspective in decision-making, and overall well-being. Previous studies have examined these age-related factors [38,39] as well as beliefs about cervical cancer and screening among women [35,38,39]. A systematic review identified women’s age as a significant predictor of attitudes toward cervical cancer screening [40]. However, the significant correlations observed between sociodemographic factors (e.g., monthly income and age) and knowledge or attitudes should be interpreted cautiously. Given the modest sample size of 60 participants, the multiple comparisons inherent in the Spearman correlation analyses increase the risk of type I error. Future studies should seek to replicate these demographic associations in larger, and potentially longitudinal, samples.

Finally, our finding that women who were aware of Pap tests had better knowledge about cervical cancer and the screening process is supported by similar studies [26,37]. This association may reflect prior exposure to health information from a range of sources. If that information is accurate, it may help women better understand the disease and the purpose of screening.

A notable limitation of this study is that the one-group pre-post design inherently limits causal inference, making it difficult to attribute the observed improvements solely to the intervention because external factors or pretest exposure may also have influenced the results. In addition, pretest exposure itself may have affected post-intervention scores. Furthermore, reliance on a self-administered questionnaire introduces the possibility of self-report bias and social desirability bias, whereby participants may overreport positive attitudes or engagement in desirable health behaviors. Critically, the short 4-week follow-up period limits assessment of sustained behavioral change, including actual screening uptake, meaning that the long-term practical impact of the intervention remains unknown. Longitudinal and comparative studies are recommended to determine whether such interventions reduce fear, embarrassment, and other barriers to screening uptake and to assess actual Pap test rates over 6 to 12 months.

Nevertheless, to our knowledge, this is the first study in Jordan to assess the effect of a 4-week, 10-session educational intervention on women’s knowledge and attitudes toward cervical cancer screening in primary healthcare centers. The intervention effectively improved knowledge and attitudes toward screening. These findings underscore the important role of education in promoting informed attitudes and preventive behaviors.

These findings may be used by nurses and midwives to guide community-based, culturally appropriate educational efforts aimed at dispelling misconceptions, sharing reliable resources, and promoting early detection. Educational programs should be intensified for specific sociodemographic groups, such as lower-income, younger, and unemployed women, who demonstrated lower knowledge and less favorable attitudes. Future interventions should also incorporate specific strategies to address deeply rooted psychological barriers, including embarrassment and fear of results. In addition, these findings may inform policy efforts to integrate standardized, culturally sensitive educational modules on cervical cancer screening into routine primary healthcare services in the absence of a national screening program.

## Figures and Tables

**Table 1.** Session content of the intervention

Session	Topics	Key contents	Intervention details (60 minutes)
1	Orientation and baseline assessment	Program overview; cervical cancer burden; baseline questionnaire; establishing group norms	• 5 min welcome/icebreaker
			• 10 min short lecture (program and burden)
			• 15 min baseline questionnaire
			• 15 min group discussion (norms and expectations)
			• 10 min Q & A
			• 5 min recap/close
2	Cervical anatomy and HPV	Basic anatomy; HPV infection and transmission; relationship to cervical cancer	• 5 min recap
			• 15 min short lecture (anatomy and HPV basics)
			• 15 min case scenario
			• 15 min group discussion (myths vs. facts)
			• 8 min Q & A
			• 2 min key messages
3	Risk factors and symptoms	Behavioral and reproductive risk factors; warning signs; myths and misconceptions	• 5 min recap
			• 15 min short lecture
			• 20 min case scenarios (small groups)
			• 10 min group discussion (myths)
			• 8 min Q & A
			• 2 min recap
4	Prevention and HPV vaccination	Primary prevention; HPV vaccination basics; eligibility; addressing concerns	• 5 min recap
			• 15 min short lecture (prevention and vaccination)
			• 20 min role-play (address concerns)
			• 10 min group discussion
			• 8 min Q & A
			• 2 min recap
5	Screening overview	Purpose of screening; who should be screened; recommended intervals; available tests	• 5 min recap
			• 20 min short lecture (screening overview)
			• 15 min group activity (match age with test)
			• 10 min group discussion (barriers)
			• 8 min Q & A
			• 2 min recap
6	Pap test procedure	Step-by-step Pap test process; what to expect; addressing pain and safety concerns	• 5 min recap
			• 20 min demonstration and short lecture
			• 15 min Q & A (fears/concerns)
			• 15 min skills practice (how to talk to provider)
			• 5 min recap
7	Results and follow-up	Understanding normal/abnormal results; follow-up pathways; treatment of pre-cancer	• 5 min recap
			• 15 min short lecture
			• 20 min case scenarios (results then move to next step)
			• 10 min group discussion
			• 8 min Q & A
			• 2 min recap
8	Barriers and coping strategies	Embarrassment, fear, and stigma; practical solutions; communicating with providers	• 5 min recap
			• 10 min guided discussion (normalize barriers)
			• 20 min group work (barriers and solutions mapping)
			• 15 min role-play (communication)
			• 8 min Q & A
			• 2 min recap
9	Building self-efficacy and support	How to request screening; involving family support; planning for screening	• 5 min recap
			• 10 min short lecture (self-efficacy)
			• 25 min action-planning worksheet
			• 15 min buddy discussion and role-play (family support)
			• 5 min recap
10	Review and post-test	Key messages recap; addressing remaining questions; post-test questionnaire; take-home materials	• 5 min recap
			• 20 min review game/Q & A
			• 15 min post-test
			• 15 min discussion (correct answers and clarify)
			• 5 min closing

HPV: Human papillomavirus; Pap test: Papanicolaou test.

**Table 2.** Characteristics of the study participants (N=60)

Items	Categories	Value
Working status	Working	21 (35.0)
	Not working	39 (65.0)
Marital status	Married	59 (98.3)
	Widowed	1 (1.7)
Health insurance	Yes	52 (86.7)
	No	8 (13.3)
Family planning used	Yes	40 (66.7)
	No	20 (33.3)
Pap test awareness	Yes	31 (51.7)
	No	29 (48.3)
Family history of cervical cancer	Yes	3 (5.0)
	No	57 (95.0)
Pap test intention	Yes	6 (10.0)
	No	54 (90.0)
Preferred provider for Pap test	Male doctor	0 (0)
	Female doctor	50 (83.3)
	No differences	10 (16.7)
Time since last gynecologist visit	One month ago	23 (38.3)
	More than 1 month ago	17 (28.3)
	Never	20 (33.3)
Educational level	Primary	6 (10.0)
	Secondary	16 (26.7)
	College/University	38 (63.3)
Number of births	0	3 (5.0)
	1	9 (15.0)
	2–3	21 (35.0)
	≥4	27 (45.0)
Age (year)		34.88±6.33 (25–45)
Age at first pregnancy (year)		22.83±3.55 (16–32)
Age at menarche (year)		14.01±1.52 (11–17)
Monthly income (JD)		388.66±188.37 (100–1,000)

Values are presented as n (%) or mean±SD (range). JD: Jordanian Dinar (1 JD is approximately 1.41 USD); Pap test: Papanicolaou test.

**Table 3.** Wilcoxon signed-rank test for the effectiveness of the program on women’s knowledge and attitudes toward cervical cancer (N=60)

Variables	n	Mean rank^†^	Median		*Z*	*p*	Cohen’s d
			Pre	Post			
Knowledge	59	30.98	3.00	16.00	–6.73	<.001	0.87
Attitudes	57	29.99	27.00	36.00	–6.62	<.001	0.85

† The mean rank is based on negative ranks.

**Table 4.** Differences in knowledge by study participants’ characteristics (N=60)

Variables	Categories	Mean rank	U^†^ or *χ*^2^^‡^ or rho^§^	Z	*p*
Working status	Working	33.07	355.50^†^	–0.84	.339
	Not working	29.12			
Educational level	Primary	22.17	3.46^‡^		.178
	Secondary	26.41			
	Tertiary	33.54			
Health insurance	Yes	29.45	153.50^†^	–1.20	.232
	No	37.31			
Pap test awareness	Yes	35.10	307.00^†^	–2.13	.033
	No	25.59			
Pap test intention	Yes	27.58	144.50^†^	–0.44	.664
	No	30.82			
Age			0.15^§^		.246
Monthly income			0.28^§^		.031

Pap test: Papanicolaou test. Significance statistics for which rho is often used include

† Mann‒Whitney U test,

‡ Kruskal‒Wallis H statistic, and

§ Spearman correlation coefficient (rho).

**Table 5.** Differences in attitudes by study participants’ characteristics (N=60)

Variables	Categories	Mean rank	U^†^ or *χ*^2^^‡^ or rho^§^	Z	*p*
Working status	Working	39.19	227.00^†^	–2.92	.004
	Not working	25.82			
Educational level	Primary	22.00	1.72^‡^		.423
	Secondary	30.69			
	Tertiary	31.76			
Health insurance	Yes	30.47	206.50^†^	–0.03	.973
	No	30.69			
Pap test awareness	Yes	30.98	434.50^†^	–0.23	.819
	No	29.98			
Pap test intention	Yes	25.08	129.50^†^	–0.83	.409
	No	31.10			
Age			0.30^§^		.020
Monthly income			0.43^§^		.001

Pap test: Papanicolaou test. Significance statistics for which rho is often used include

† Mann‒Whitney U test,

‡ Kruskal‒‎Wallis H statistic, and

§ Spearman correlation coefficient (rho).
